# Polygenetic-Risk Scores Related to Crystallin Metabolism Are Associated with Age-Related Cataract Formation and Interact with Hyperglycemia, Hypertension, Western-Style Diet, and Na Intake

**DOI:** 10.3390/nu12113534

**Published:** 2020-11-17

**Authors:** Donghyun Jee, Suna Kang, ShaoKai Huang, Sunmin Park

**Affiliations:** 1 Division of Vitreous and Retina, Department of Ophthalmology, St. Vincent’s Hospital, College of Medicine, The Catholic University of Korea, Suwon 16247, Korea; doj087@mail.harvard.edu; 2 Food and Nutrition, Obesity/Diabetes Research Center, Institute of Basic Science, Hoseo University, Asan 31499, Korea; roypower003@naver.com (S.K.); huangsk0606@gmail.com (S.H.)

**Keywords:** Age-related cataract, crystalline, genetic impact, hypertension, hyperglycemia, coffee intake

## Abstract

Age-related cataract (ARC) development is associated with loss of crystalline lens transparency related to interactions between genetic and environmental factors. We hypothesized that polygenetic risk scores (PRS) of the selected genetic variants among the ARC-related genes might reveal significant genetic impacts on ARC risk, and the PRS might have gene–gene and gene–lifestyle interactions. We examined the hypothesis in 1972 and 39,095 subjects aged ≥50 years with and without ARC, respectively, in a large-scale hospital-based cohort study conducted from 2004 to 2013. Single nucleotide polymorphisms (SNPs) of the genes related to ARC risk were identified, and polygenetic risk scores (PRS) were generated based on the results of a generalized multifactor dimensionality reduction analysis. Lifestyle interactions with PRS were evaluated. The PRS derived from the best model included the following six SNPs related to crystallin metabolism: *ULK4*_rs1417380362, *CRYAB*_rs2070894, *ACCN1*_rs55785344, *SSTR2*_rs879419608, *PTN*_rs322348, and *ICA1*_rs200053781. The risk of ARC in the high-PRS group was 2.47-fold higher than in the low-PRS group after adjusting for confounders. Age, blood pressure, and glycemia interacted with PRS to influence the risk of ARC: the incidence of ARC was much higher in the elderly (≥65 years) and individuals with hypertension or hyperglycemia. The impact of PRS on ARC risk was greatest in middle-aged individuals with hypertension or hyperglycemia. Na, coffee, and a Western-style diet intake also interacted with PRS to influence ARC risk. ARC risk was higher in the high-PRS group than in the low-PRS group, and high Na intake, Western-style diet, and low coffee intake elevated its risk. In conclusion, ARC risk had a positive association with PRS related to crystallin metabolism. The genetic impact was greatest among those with high Na intake or hypertension. These results can be applied to precision nutrition interventions to prevent ARC.

## 1. Introduction

A cataract is defined as the loss of crystalline lens transparency, which is induced by changes in lens structure and its proteins, that result in light scattering [1]. Cataracts are classified as congenital, presenile, and senile (age-related) according to the age of onset. The etiologies of congenital cataracts and age-related cataracts (ARC) are not the same. A congenital cataract is often associated with deterioration of lens microstructure, whereas ARC usually involves the deposition of high molecular weight protein aggregates (>1000 Å) that disrupt the micro-crystalline lens structure [1]. However, all cataracts are caused by protein aggregates, protein insolubility, and covalent cross-linking of lens proteins [2]. ARC typically develops in adults aged ≥ 50 years and is usually corrected surgically. Despite surgical advances, ARC remains a primary cause of visual impairment and is responsible for causing blindness in 35% of the blind population aged ≥ 50 years worldwide [3]. Moreover, in low- and middle-income countries, ARC remains the leading cause of blindness [3].

Genetic, environmental, and diet-related risk factors contribute to the risk of developing ARC. The identified modifiable risk factors include obesity, hypertension, hyperglycemia, smoking, ultraviolet exposure, and low intakes of vitamin C, carotenoids, lutein, zeaxanthin, or n-3 fatty acids [4]. These risk factors are related to the modulation of oxidative stress caused by exposure to reactive oxygen species (ROS) and inflammation. Obesity, hypertension, and hyperglycemia elevate oxidative stress and inflammation during endogenous energy production in mitochondria, whereas smoking and UV exposure are exogenous causes of oxidative stress [5]. On the other hand, phytochemicals and vitamins A and C eliminate ROS and decrease oxidative stress; thereby lowering the risk of ARC [5]. Although genetic factors are generally considered immutable, the genetic impacts can have an interaction with environmental factors [4]. Some genetic variants of genes encoding crystallin proteins and antioxidant enzymes have been reported to contribute to the underlying mechanisms of ARC, but the reported results were only marginally significant (*p* = 0.05) or non-significant [3,5,6].

Genetic factors related to inherited cataracts are mainly associated with genes encoding crystallins, and some of the same genes are also associated with ARC [7]. Polymorphisms in the promotor of crystallin Alpha A (*CRYAA*; rs7278468 and rs13053109) increase ARC risk by reducing the transcription of *CRYAA*, which is involved in lens transparency and opacity [8]. Furthermore, the rs1801133 polymorphism of methylenetetrahydrofolate reductase (*MTHFR*) exhibits a significant association with ARC. The minor MTHFR T allele of rs1801133 has a positive association with ARC risk (OR = 1.26, *p* = 0.003) and the s3737967, rs1801131, rs1801133 haplotypes (OR = 1.55, *p* = 0.003) and *MTHFR* rs9651118 are also linked to ARC risk [9]. Interestingly, *MTHFR* is involved in homocysteine metabolism and also modulates serum concentrations of homocysteine—a potential risk factor for insulin resistance. The minor allele rs1801133 of *MTHFR* decreases its activity and may increase serum homocysteine concentrations, which would increase the risk of both ARC and metabolic syndrome [9]. These results suggest that genetic risk factors for metabolic syndrome, as well as dietary and environmental factors that exacerbate the genetic risk factors, may be important risk factors for ARC. However, the influences of environmental and diet-related factors on the genetic risk factors for ARC risk have not been studied. The majority of studies on genetic variants in ARC have been conducted on single SNPs or haplotypes and reported only minor impacts on ARC risk. However, we hypothesized that polygenetic risk scores (PRS) of selected genetic variants in the ARC-related genes, identified by logistic regression, might reveal significant genetic impacts on ARC risk. Here, we investigated the polygenetic variants that confer ARC risk and the additional effects of gene–gene and gene–lifestyle interactions in middle-aged and elderly participants.

## 2. Materials and Methods

### 2.1. Participants

During 2004–2013, 58,630 Korean adults aged >40 years volunteered to participate in a hospital-based city cohort study called the Korean Genome and Epidemiology Study (KoGES) organized by the Korean Centers for Disease Control and Prevention. All procedures of the KoGES were under the Declaration of Helsinki and approved by the Institutional Review Board of the Korean National Institute of Health (KBP-2015-055). The present study had additional approval by the Institutional Review Board of Hoseo University (1041231-150811-HR-034-01). All participants provided written informed consent.

### 2.2. Criteria of ARC

The participants aged ≥50 years (*n* = 39,095) were included to explore the genetic impact of ARC. Participants were asked whether they had a diagnosis of ARC, and those that had a positive answer were considered to have the condition (*n* = 1972).

### 2.3. Demographic, Anthropometric, and Biochemical Information

Participants were interviewed to obtain demographic information (e.g., age, education, income, smoking history, alcohol consumption, and physical activity [10]. Household income was stratified as very low (<USD 1000/month), low (USD 1000–2000/month), intermediate (USD 2000–4000/month), or high (>USD 4000/month) [11]. Education level was classified as lower than high school, high school, or college or higher. The participants were categorized by alcohol consumption into no (0 g), mild (0–20 g; equivalent to 0–400 mL beer), or moderate (>20 g; the equivalent of about 400 ml beer) drinkers based on average daily alcohol consumption estimated from the amounts of different alcoholic beverages consumed and their typical alcohol contents (Table 1) [11]. Smoking status was defined as current when > 100 cigarettes had been smoked over the previous six months and categorized as current, past, or never [11]. Three dietary patterns clustered by the principal component analysis (PCA) method were categorized as a balanced dietary pattern, Western-style dietary pattern, and rice-based dietary pattern. Each dietary pattern was divided into low and high intake by the 70th percentile of its intake.

Anthropometric parameters (body weight, height, and waist circumference) were assessed using a standardized procedure [12]. Body mass index (BMI) was calculated by dividing weight (kilograms) by height^2^ (meters). Blood pressure was measured on right arms, in a sitting position, at heart level. Biochemical parameters were determined using plasma and serum from blood drawn after an overnight fast [12]. Fasting serum glucose and blood hemoglobin A1c (HbA1c; glycated hemoglobin) concentrations were determined using a Hitachi 7600 Automatic Analyzer (Hitachi, Tokyo, Japan), and plasma lipid profiles (total cholesterol, high-density lipoprotein (HDL), and triglyceride) using a Hitachi 7600 Automatic Analyzer.

### 2.4. Food and Nutrient Intake Assessments

Food and nutrient intakes were determined using a semi-quantitative food frequency questionnaire (SQFFQ) developed and validated during the KoGES [13]. Usual dietary intake over the previous six months was estimated for each participant by using the SQFFQ. The questionnaire requested information regarding the consumption of 103 food items. The 23 nutrient intakes from the SQFFQ data were calculated using the Computer-Aided Nutritional Analysis Program (CAN Pro) 3.0, a nutrient database program developed by the Korean Nutrition Society [13].

The 103 food items in the SQFFQ were categorized into 29 predefined food groups [14,15]. The food groups were used as independent variables during factor analysis conducted to find dietary patterns using the FACTOR procedure. We determined the number of factors to retain using eigenvalues of >1.5 and 4 dietary factors describing the distinct dietary patterns of participants. The orthogonal rotation procedure (varimax) was applied during principal component analysis [14]. Factor-loading values ≥0.40 were considered to make primary contributions to distinct dietary patterns (Supplemental Table S1).

### 2.5. Genotyping Using a Korean Chip and Quality Control

Genotype data were provided by the Center for Genome Science at the Korea National Institute of Health. Genomic DNA was isolated from whole blood, and genotypes were determined using a Korean Chip (Affymetrix, Santa Clara, CA, USA), which was designed to study Korean genetic variants and included disease-related SNPs [16]. Genotyping accuracy was checked by Bayesian Robust Linear Modeling using the Mahalanobis Distance Genotyping Algorithm [16]. For the genotyping analysis by this Korean chip, genotyping accuracy was ≥98%, the missing genotype call rate was <4%, heterozygosity was <30%, and it showed no gender bias. Genetic variants that met the Hardy–Weinberg equilibrium (HWE, *p* > 0.05) criterion were included [16].

### 2.6. Genetic Variants Influencing ARC Risk and the Best Model for Detecting Gene–Gene Interactions as Determined by Generalized Multifactor Dimensionality Reduction (GMDR)

Participants aged ≥ 50 years were dichotomized into ARC (case, *n* = 1972) and non-ARC (*n* = 39,095) groups. We conducted a logistic regression for the 55 genes related to cataract to identify genetic variants associated with ARC risk and selected those with *p* < 0.0001. Linkage disequilibrium (LD) analyses were performed on selected SNPs in the same chromosomes using Haploview 4.2 in PLINK. Since SNPs with high D’ values ((D’ ≥ 0.3) provided the same information on genetic impact, they were excluded in the generalized multifactor dimensionality reduction (GMDR). 

Of the 35 potential genetic variants, those exhibiting a gene–gene interaction associated with ARC risk were selected by GMDR [17,18,19]. The best gene–gene interaction model was searched for by using a sign rank test of trained balanced accuracy (TRBA) and testing balanced accuracy (TEBA), with or without adjusting for covariates using a GMDR program and a *p*-value threshold of 0.05 [19]. The covariates used were age, gender, residence area, education, income level, and body mass index. Ten-fold cross-validation was also checked by cross-validation consistency (CVC) since the sample size was over 1000 [19]. Therefore, a 10 out of 10 for CVC met the criteria for perfect cross-validation. Using the best model determined by GMDR analysis, the risk allele of each SNP was designated with a value of 1 [10]. If a person had AA, AG, or GG of one SNP, and the A allele was the risk allele, the genetic score for the SNP was 2, 1, or 0. The polygenetic risk score for the best gene–gene interaction model (PRS) was assessed by a summation of the number of the risk alleles (genetic score) from each selected SNP in the best gene–gene interaction model. PRS was divided into three categories by tertiles (low-PRS, middle-PRS, and high-PRS).

### 2.7. Statistical Analyses

Statistical analysis was conducted using PLINK version 2.0 (http://pngu.mgh.harvard.edu/~purcell/plink) and SAS version 9.3 (SAS Institute, Cary, NC, USA). Descriptive statistics of categorical variables (e.g., gender and smoking status) were analyzed using frequency distributions in the low-, middle-, and high-PRS groups. Statistical differences between their frequency distributions were assessed using the chi-squared test. The descriptive values of continuous variables were provided by means and standard deviations, according to the PRS categories. The significance of differences between PRS groups was analyzed by one-way analysis of variance (ANOVA) with adjustment for covariates. Multiple comparisons between PRS groups were performed using Tukey’s test.

The association between PRSs obtained using the best model, and ARC risk was examined using multivariate logistic regression analysis with adjustment for covariates. Adjusted odds ratios (ORs) and 95% confidence intervals (CI) for ARC risk were calculated using low-PRS as the reference. Multivariate logistic regression analysis was performed using two adjusted models. Covariates of model 1 were sex, age, residence area, BMI, and education and income levels. Model 2 included covariates of sex, age, residence area, BMI, education and income levels, smoking status, drinking status, daily energy intake, total physical activity and arthritis, and use of dermatitis medicine.

In determining the interaction between PRSs and demographic and dietary intake parameters, the participants were categorized into higher and lower intake groups using the classification criterion described above. A multivariate interaction model was used to examine interactions between PRS and lifestyles and demographic parameters after adjusting for covariates. Statistical significance was accepted for *p*-values < 0.05.

## 3. Results

### 3.1. Demographic, Anthropometric, and Biochemical Parameters According to Gender and Cataract Incidence

Participants of both genders with ARC were older than those without ARC. BMI and waist circumferences were not associated with cataracts, although women had lower BMIs and smaller waist circumferences than men (Table 1). Participants with ARC had higher serum glucose concentrations and HbA1c concentrations in a fasted state than those without ARC. Men exhibited higher serum glucose concentrations than women. Serum total cholesterol, HDL, and low-density lipoprotein (LDL) concentrations were higher in women than men, but serum triglyceride concentrations in men and women were similar (Table 1). However, lipid profiles were not significantly different between those with or without cataracts. Both men and women with hypertension had a higher cataract incidence. Metabolic syndrome occurrence was influenced by gender and associated with the presence of ARC. Women had a slightly but significantly higher incidence of metabolic syndrome than men, and participants with metabolic syndrome had a much higher incidence of cataract than those without metabolic syndrome (Table 1). Education, income, regular exercise, and alcohol intake were significantly different by gender, but not by the presence of ARC. Smoking status and coffee drinking were related to both gender and the presence of cataract (Table 1).

### 3.2. The Best SNP Model Selected from Genetic Variants Related to ARC by Logistic Regression Analysis

Ten genetic variants were selected for potential gene–gene interactions from the 35 genetic variants associated with ARC risk by logistic regression analysis. Each SNP had a significant association with ARC risk with a *p* < 0.00001 (Table 2). The rs117418426, rs147082589, rs553983141, rs117583209, and rs55785344 SNPs exhibited a positive association with ARC risk (OR = 1.21–1.68), whereas those of rs1417380362, rs200053781, rs322348, rs2070894, and rs879419608 were negatively associated with ARC risk (OR = 0.70–0.86). All SNPs satisfied the HWE at *p* > 0.05, and minor allele frequency (MAF) values of all SNPs were greater than 0.01 (Table 2). The ten SNPs selected were rs1417380362_*unc-51 like kinase 4* (*ULK4*), rs117418426_*glutathione peroxidase 3* (*GPX3*), rs200053781_ *islet cell autoantigen 1* (*ICA1*), rs147082589_*brain-specific angiogenesis inhibitor-associated protein 2-like 1* (*BAIAP2L1*), rs322348_*pleiotrophin* (*PTN*; *neurite growth-promoting factor 1*), rs553983141_*spectrin alpha chain* (*SPTAN1*), rs117583209_*neuralized E3 ubiquitin-protein ligase 1* (*NEURL1*), rs2070894_ *crystallin alpha B* (*CRYAB*), rs55785344_*amiloride-sensitive cation channel 1*, *neuronal* (*ACCN1*), and rs879419608_ *somatostatin receptor 2* (*SSTR2*) (Table 2). All genetic variants were related to the synthesis or degradation of crystallin or neuronal proteins.

Two PRS models for gene–gene interactions, which included five or six genetic variants each, satisfied our statistical criteria after adjusting for age, gender, survey year, residence area, and BMI (*p* = 0.001 by the signed *t*-test; CVC = 10 out of 10) (Table 3). The gene–gene interaction PRS model with five SNPs included *ULK4*_rs1417380362, *CRYAB*_rs2070894, *ACCN1*_rs55785344, *SSTR2*_rs879419608, and *PTN*_rs322348; while the PRS model with six SNPs added *ICA1*_rs200053781 to the five SNPs in the first PRS model. The PRS model with five SNPs had a TRBA of 0.5581 and a TEBA of 0.5247, while the PRS model containing six SNPs had a TRBA of 0.5673 and a TEBA of 0.5292, which showed that both PRS models had significant genetic impacts on ARC risk (Table 3).

### 3.3. Association of the PRS of the Two Models with ARC Risk

**Figure 1:**
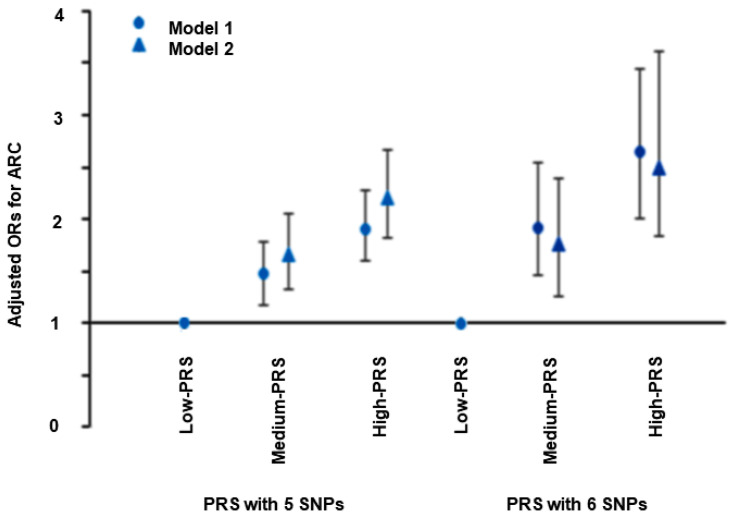
Adjusted odds ratios and 95% confidence intervals for age-related cataract (ARC) risk by polygenetic risk scores (PRS) using the 5 SNPs and 6 SNPs. PRS was calculated by the summation of polygenetic-risk scores of the best PRS model with 5 and 6 SNPs and categorized into three groups (0–6, 7–9, and ≥10) by the tertiles (low-PRS, medium-PRS, and high-PRS). They were adjusted with covariates for model 1, including age, gender, residence area, survey year, education, job, and income, for model 1 and covariates in model 2 containing for model 1 plus smoking, alcohol intake, physical activity, hypertension, serum glucose concentrations, HbA1c contents, energy intake, fat, protein and carbohydrate percent intake, and arthritis and dermatitis medicine intake for model 2.

As determined using the PRS model with five SNPs, having a high-PRS was positively associated with ARC risk by 1.89-fold (CI: 1.61–2.23) in model one and 2.15-fold (CI: 1.72–2.67) in model two (*p* < 0.001) as compared to those with a low-PRS after adjusting for the covariates. In the six SNP PRS model, having a high-PRS was positively related with ARC by 2.62-fold (CI: 1.97–3.49) in model one and 2.47-fold (CI: 1.73–3.54) in model two, compared to the low-PRS, after adjusting for the covariates (*p* < 0.001; Figure 1). These results showed that the six SNP PRS model exhibited a greater genetic impact on ARC incidence than the five SNP PRS model, and thus, was used to investigate interactions between genetic variant-metabolic parameters and genetic variant lifestyles. PRS was not associated with obesity, hyperglycemia, hypertension, or dyslipidemia (Supplemental Table S2).

### 3.4. Interaction between PRS and Metabolic Parameters and Lifestyles

**Figure 2:**
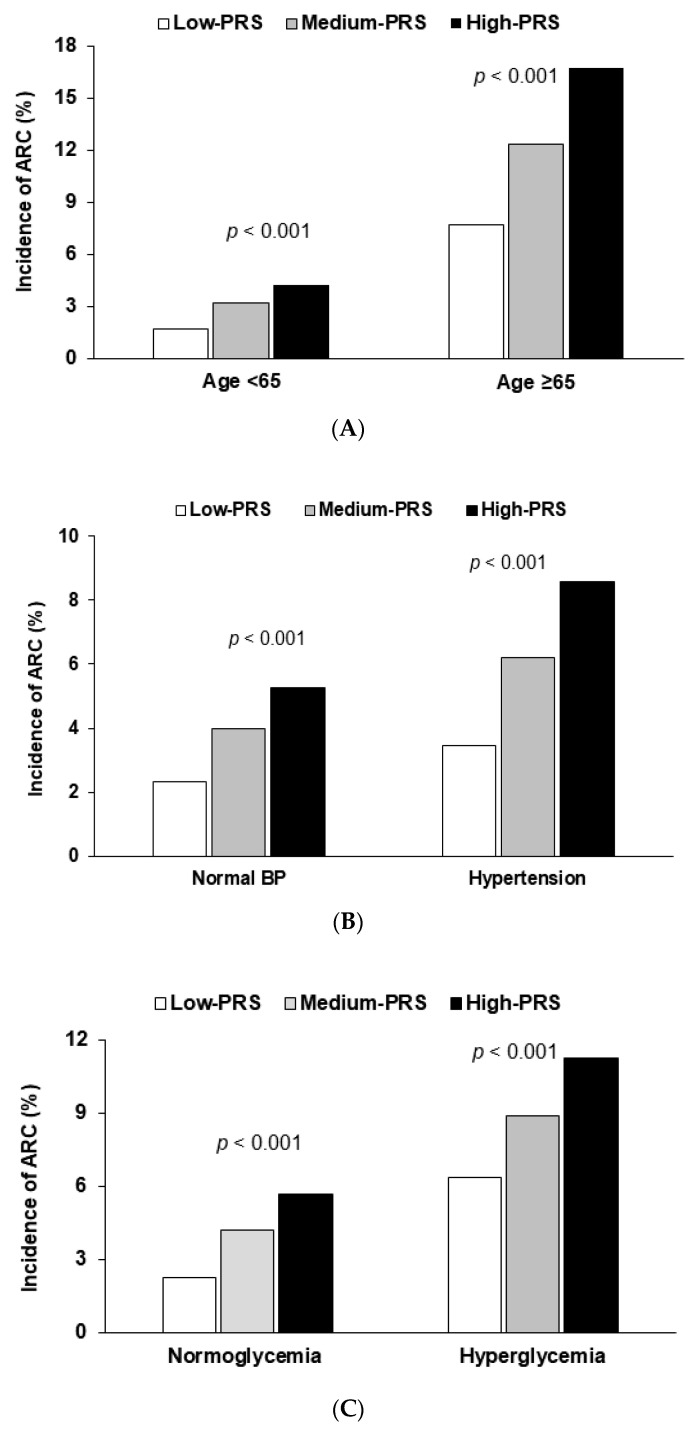
Age-related cataract (ARC) incidence among participants in the low-, medium- and high-PRS groups (defined using the 6 SNP genetic variant–genetic variant interaction model) for metabolic parameters. (**A**) In the participants, according to age (cutoff point: 65 years old). (**B**) In the participants, according to the blood pressure (cutoff point: 130 mmHg for SBP and 90 mmHg for DBP). (**C**) In the participants, according to serum glucose concentrations (cutoff point: 126 mg/dL serum glucose concentrations). PRS was calculated by the summation of polygenetic-risk scores of the best model with 6 SNPs and categorized into three groups (0–6, 7–9, and ≥ 10) by the tertiles (low-PRS, medium-PRS, and high-PRS). *p*-values represent the significance of differences among the PRS groups by χ2 test in each category.

ARC risk was influenced by age (*p* < 0.0001), but not by gender (Table 4). ARC incidence was higher in the order of low-PRS, medium-PRS, and high-PRS in middle-aged and elderly participants, but the incidence of ARC was much higher in the elderly (Figure 2A). The adjusted ORs for ARC risk were higher for middle-aged participants (ORs = 2.92) than the elderly (ORs = 2.03), which indicated that genetic impact was greater in middle-aged individuals than in the elderly. Metabolic syndrome risk did not interact with PRS for ARC risk, but interestingly, ARC risk had a positive association with high-PRS only in participants without metabolic syndrome, which indicated that metabolic syndrome offset genetic impact (*p* < 0.05; Table 4). However, the ARC incidence interacted with blood pressure (*p* = 0.03) and glucose control (*p* = 0.04) (Table 4). Although PRS had a positive association with cataract risk in participants with either normotension or hypertension, ARC risk was greater in normotensive than in hypertensive participants in the high-PRS group (Table 4). However, the prevalence of ARC increased more rapidly in hypertensive participants in the high-PRS group (Figure 2B). Serum glucose concentrations interacted with PRS in terms of ARC risk: adjusted ORs had a positive and significant association with PRSs only in those with a low serum glucose concentration (Table 4). The ARC incidence was much higher in the hyperglycemic participants with the low-PRS than in the normoglycemic participants with the low-PRS (Figure 2C). These results indicated that hyperglycemia offset the genetic impact.

**Figure 3:**
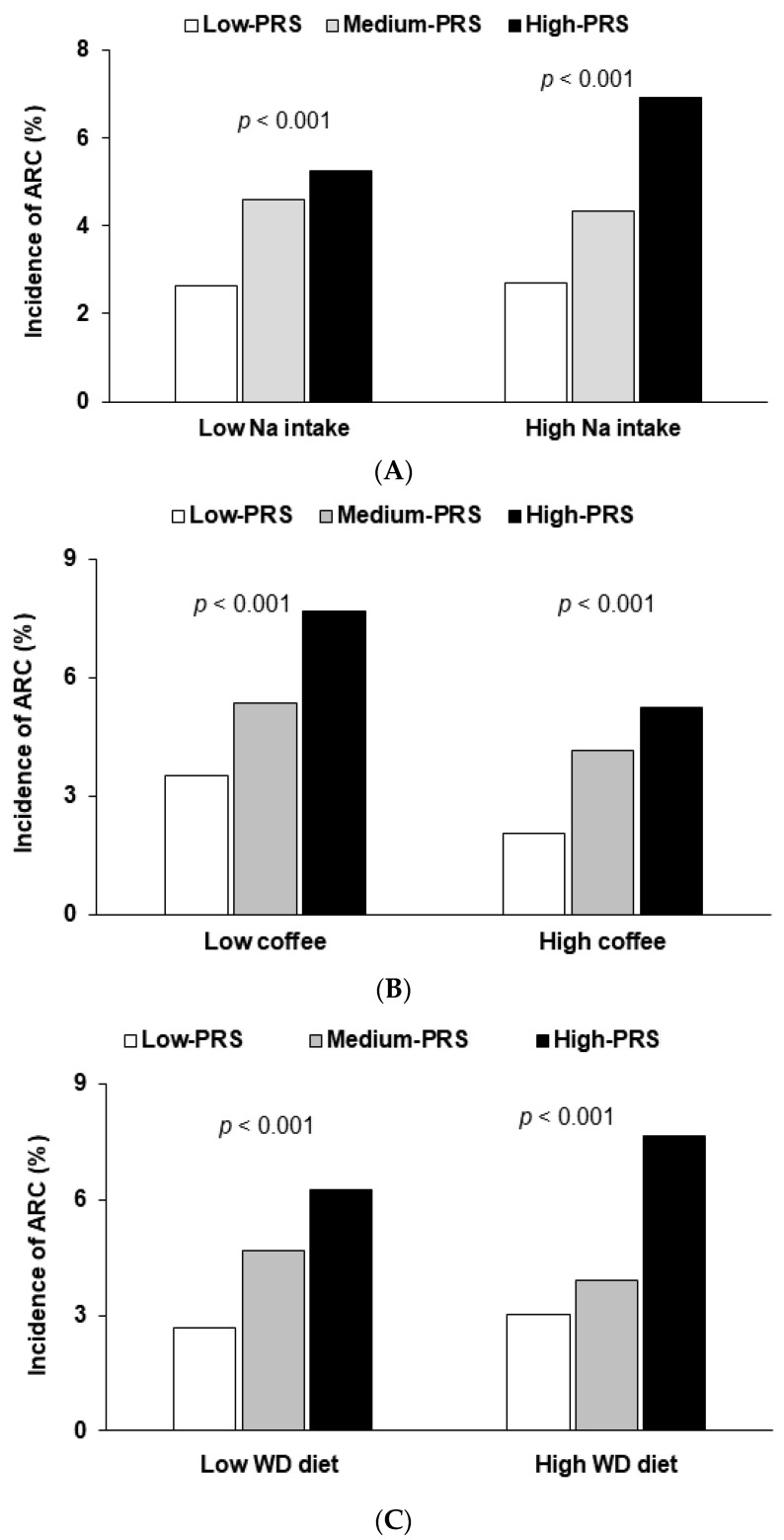
Prevalence of age-related cataracts (ARC) in participants in the low-, medium- and high-PRS groups (defined using the 6 SNP genetic variant–genetic variant interaction model): (**A**) In the participants according to the Na intake (cutoff point: 1600 mg/1000 kcal; (**B**) In the participants according to the coffee intake (cutoff point: ≥ 3 g/day); (**C**) In the participants according to the consumption of a Western-style diet pattern (WD; cutoff point: 70th percentile). PRS was calculated by the summation of polygenetic-risk scores of the best model with 6 SNPs and categorized into three groups (0–6, 7–9, and ≥ 10) by the tertiles (low-PRS, medium-PRS, and high-PRS). *p*-value represented the significant differences among the PRS groups by χ2 test in each category.

Among the interactions of PRS with food intake, Na, coffee, and Western-style diet intakes had interactions with the PRS (Table 5). Adjusted ORs of ARC risk in the high-PRS group were higher in participants with high Na intake (Figure 3A), indicating that low Na intake protects against ARC in individuals with a high-PRS. Adjusted ORs for ARC risk in the high-PRS group were higher for participants with low coffee intake and a high Western-style diet intake (Table 5). For those with high coffee intake, the incidence of ARC was much lower than for those with low consumption, and ARC incidence increased in the order of low-PRS, medium-PRS, and high-PRS (Figure 3B). Thus, coffee intake (>3 g/day) might protect against ARC risk in people with a high-PRS. However, a high Western-style diet intake with high noodle, bread, and meat intakes, increased ARC incidence as compared with their low consumption. The impact of PRS in ARC risk was higher in those with a high Western-style diet intake (Figure 3C; Table 5).

## 4. Discussion

In the present study, we constructed a PRS for ARC risk by using a generalized multifactor dimensionality reduction analysis and evaluated whether the PRS was associated with the presence of cataracts. We also assessed genetic variant–metabolic syndrome and genetic variant–diet interactions. The PRS included six SNPs related to crystallin metabolism. We found that the risk of ARC was 2.47 times higher for a high-PRS than a low-PRS after adjusting for covariates. Furthermore, the genetic risk of ARC development was found to be modified by age, hypertension, serum glucose, sodium and coffee intake, and a Western-style diet. This is the first study to show that an unfavorable genetic profile can greatly increase the risk of cataracts.

Several genetic variants related to crystallin metabolism were selected for the association with ARC in this study. The apoptosis of lens epithelial cells is associated with the development of cataracts. *GPX3* can downregulate cataracts formation by upregulating glutathione peroxidase 3, which decreases the oxidative stress in the anterior capsular epithelial cells [20]. The *CRYAB* gene encodes alpha-B crystallin protein, and mutations of *CRYAB* cause posterior polar cataracts, which is a distinct clinical type of cataracts [21]. The *SSTR* gene encodes the human somatostatin receptor, and its mutation may be responsible for the development of cerulean cataracts. Cerulean cataracts is characterized by variable coarse deposits, mainly in the lamella, and it has a combination of white, blue, and purple hues [22]. The *ACCN1* gene encodes amiloride-sensitive cation channel neuronal 1, which is one of the plasma membrane channel proteins. The mutation of this gene may result in a dysfunction of lens epithelial cells. *ULK4*, which encodes serine/threonine-protein kinase, may be involved in the remodeling of cytoskeletal components, such as alpha-tubulin. However, none of the individual genetic variants could explain the development of ARC, which is one of the reasons why we constructed the PRS.

This study revealed that the adjusted OR for ARC in high-PRS participants was higher for middle-aged participants than in the elderly participants, which was unexpected. Age has been identified as the most predominant risk factor of ARC development in various epidemiologic studies [23,24], and several Korean epidemiologic studies have reported a linear association between ARC incidence and age [25,26]. These results suggest that people at greater genetic risk are especially susceptible to early-onset ARC.

Hypertension also interacted with the genetic risk of ARC, which was significant for participants with or without hypertension, but the effect of genetic risk factors was greater in those with hypertension in the present study. A meta-analysis of 25 studies confirmed that the risk of ARC in individuals with hypertension was 1.08 times higher in cohort studies and 1.28 times higher in cross-sectional and case–control studies [27]. Hypertension is associated with oxidative damage and xanthine oxidase, myeloperoxidase, and glutathione peroxidase induced lipid peroxidation of crystalline lens proteins [27]. Our findings indicate that hypertension might increase the genetic risk of ARC development.

Serum glucose levels also modified the genetic risk of ARC development. The risk of ARC determined by PRS was significant only in those with a low serum glucose level (<126 mg/dL). It was interesting that subjects with a serum glucose concentration level of ≥126 mg/dL taking hypoglycemic medicine exhibited no association with additional genetic risk of ARC, given that a high glucose level is a well-known risk factor of ARC. The prospective Blue Mountains Eye Study reported that the 10-year incidence of ARC in those with a high glucose level was 1.79 times higher for a cortical cataract than for normoglycemic participants [28]. This may be because high glucose levels oxidize crystalline lens proteins and decrease superoxide dismutase activity, which leads to the aggregation of lens proteins and subsequent cataract formation [29,30]. Accordingly, high serum glucose levels may be such a high-risk factor that it decreases the relative effect of genetic risk on ARC development.

High sodium intake increased the effect of the genetic risk of ARC significantly in the present study. As determined by PRSs in participants with low sodium intake, the genetic impact did not show a linear pattern, indicating that there was no dose-dependent association. A cross-sectional epidemiology study using Korea National Health and Nutritional Examination Survey data showed that those with a high sodium intake had a 1.29 times higher prevalence of ARC and that this effect was more prominent in patients >50 years old [31]. These results suggest that a low-salt diet might help prevent ARC in the elderly, especially in those with a high-PRS, because high sodium intake may increase the genetic risk of ARC development.

Caffeine intake had an inverse association with the ARC prevalence in the present study, which was consistent with earlier studies [32,33]. A previous study proposed that coffee intake may prevent ARC formation in animal models by maintaining lens glutathione and ascorbic acid levels [32]. Another study using global data from the World Health Organization and the United Nations Food and Agriculture Organization reported that the incidence of cataract blindness was significantly lower in those consuming higher amounts of coffee [33]. Furthermore, our findings showed that coffee intake interacted with the genetic risk of ARC, decreasing the incidence of ARC even in people at high genetic risk of developing ARC.

In the present study, a Western-style diet was characterized by high intakes of bread, fast food, and meat. Participants in the >70th percentile for a Western-style dietary intake were assigned to the high Western-style diet group. Western-style diets showed a genetic interaction with ARC risk. The risk of ARC, according to PRS, was significant only in those with a high Western-style diet, whereas in those with a low Western-style diet, PRS was not a significant risk factor for ARC. A Western-style diet may increase serum glucose levels due to increasing insulin resistance, and a Western-style diet increased the effect of genetic risk factors on ARC development.

Other well-known risk factors, such as BMI, were found not to be significantly associated with the development of ARC in the present study. A meta-analysis indicated that elevated BMI might increase the risk of ARC [34]. However, in the Blue Mountains Eye Study, a longitudinal population-based study of common eye disease, there were no associations between BMI and any type of cataracts [35]. One possible explanation for the lack of association between BMI and ARC is that Asians, including Koreans, develop metabolic diseases, particularly type 2 diabetes, at much lower BMIs than Caucasians [36]. For example, a significant portion of diabetic patients are non-obese type 2 diabetics, and their body weight often decreases during the disease course [36]. Hyperglycemia is a crucial risk factor for ARC, and the opposite direction in the status of BMI and hyperglycemia might contribute to the lack of a significant association of BMI with ARC. However, further study is needed to evaluate the association between BMI and cataract in Koreans.

The present study has several strengths and limitations. The merit of this study was its relatively large number of participants—1972 patients with ARC and 39,095 subjects without ARC—which increases the reliability of the results. Moreover, lifestyle factors, including coffee consumption, sodium intake, and Western-style diets, were shown to modulate the impact of the polygenetic risk factors for ARC. These dietary risk factors contribute only small risks for people with a favorable genotype, but they greatly increase the risk of cataracts for a subgroup of people with a high PRS. The limitations of our study are as follows. First, the presence of ARC was evaluated by a questionnaire about the previous diagnosis of cataracts by a doctor. However, the subtypes of ARC were not evaluated with a slit-lamp examination. Given that not all the risk factors are equally responsible for causing ARC, the genetic impact on each type of cataracts could not be assessed. Further study is needed to examine the PRS for the different subtypes of ARC. Second, due to the case–control study, the results could not be explained as a cause-and-effect relationship. Finally, the SQFFQ method used to evaluate nutrition also has some limitations. In particular, it might underestimate or overestimate nutrition intake in each person, because it relies on participants’ memories to recall past consumption. However, the SQFFQ was developed and validated for the KoGES and has been widely used in many other studies [13,37,38].

## 5. Conclusions

We constructed a polygenetic risk scoring system for ARC by generalized multifactor dimensionality reduction and found the risk of ARC was 2.47 times higher in the high-PRS group than in the low-PRS group. The people with high-PRS accounted for about 23% of the total population. This is a much higher genetic risk than previous studies have shown by evaluating the risk attributable to individual genetic risk factors. Furthermore, the study demonstrates that genetic variant–metabolic syndrome and genetic variant–diet interactions influence the genetic risk of ARC development. More specifically, the genetic risk for ARC development was modified by age, the presence of hypertension, hyperglycemia, sodium and coffee intakes, and a Western-style diet. The take-home message is that people with known genotypes, and perhaps family histories, that put them at high risk should be careful to avoid the dietary and lifestyle factors linked to ARC, because these will probably put them at much greater risk for ARC compared to people with lower genetic risks. Given that ARC is the leading global cause of blindness, further studies should be undertaken to identify and confirm cause and effect relationships between genetic and lifestyle-related risk factors that can reduce the risk of ARC development. The mechanisms involved in dietary modulation of the risks and possibly identifying potentially beneficial dietary components need to be studied in randomized clinical trials. The results can be applied for precision medicine to protect against ARC in the person with genetic risk.

## Figures and Tables

**Table 1:** Demographic, anthropometric, and biochemical parameters according to gender and age-related cataract (ARC) incidence.

Parameters	Men		Women	
	Non-ARC (*n* = 14,806)	ARC (*n* = 805)	Non-ARC (*n* = 24,289)	ARC (*n* = 1167)
Age (years)	59.5 ± 5.7 ^c^	63.0 ± 5.6 ^a^	56.8 ± 5.3 ^d^	61.0 ± 5.2 ^b^***^+++^
BMI (kg/m^2^)	24.4 ± 2.6 ^a^	24.5 ± 2.7 ^a^	23.9 ± 2.9 ^b^	23.9 ± 3.0 ^b+++^
Waist circumferences (cm)	85.2 ± 7.3 ^a^	85.2 ± 7.3 ^a^	79.7 ± 7.9 ^b^	80.0 ± 8.0 ^b+++^
Serum glucose (mg/dL)	99.6 ± 23 ^b^	103 ± 30 ^a^	94.8 ± 18.3 ^c^	98.6 ± 23 ^b^***^+++^
HbA1c (%)	5.81 ± 0.83 ^b^	5.90 ± 0.96 ^a^	5.78 ± 0.68 ^b^	5.95 ± 0.94 ^a^***
Serum total cholesterol (mg/dL)	191 ± 35 ^b^	185 ± 37 ^c^	207 ± 36 ^a^	206 ± 38 ^a^**^+++^
Serum LDL (mg/dL)	112 ± 33 ^b^	108 ± 33 ^b^	125 ± 33 ^a^	125 ± 35 ^a+++^
Serum HDL (mg/dL)	50.0 ± 12.1 ^b^	49.9 ± 12.2 ^b^	56.9 ± 13.1 ^a^	56.3 ± 12.5 ^a+++^
Serum triglyceride (mg/dL)	142 ± 95 ^a^	134 ± 109 ^a^	122 ± 74 ^b^	127 ± 74 ^b+++^
Hypertension (%)	4935 (35.3)	351 (43.6) ^+++^	6504 (26.8)	452 (38.7) *^+++^
Metabolic syndrome (%)	2711 (19.4)	196 (24.4)	3762 (15.5)	302 (25.9) ***^+^
Education (Number, %)				
<High school High school College or more	1440 (15.8) 2104 (23.0) 5589 (61.2)	103 (20.1) 121 (23.6) 288 (56.3)	5395 (26.4) 5536 (27.1) 9487 (46.5)	377 (38.7) ^+++^ 274 (28.1) 324 (33.2)
Income (Number, %) <USD 1000/month	1405 (10.6)	111 (14.6)	3457 (15.2)	311 (28.7) ^+++^
USD 1000–2000	2962 (22.4)	197 (25.8)	5829 (25.6)	333 (30.7)
USD 2000–4000	5577 (42.1)	300 (39.3)	9272 (40.8)	341 (31.5)
USD 4000	3308 (25.0)	155 (20.3)	4172 (18.4)	99 (9.1)
Exercise (Number, %) No Yes	5472 (39.2) 8487 (60.8)	272 (34.1) 526 (65.9)	11,121 (45.9) 13,102 (54.1)	536 (46.1) ^+++^ 627 (53.9)
Smoking (Number, %) No Former smoking	4689 (33.5) 6005 (42.9)	264 (32.9) 348 (43.3)	23719 (97.7) 228 (0.94)	1144 (98.0) ***^++^ 14 (0.26)
Smoking	2250 (23.7)	192 (23.9)	332 (1.37)	9 (0.78)
Alcohol intake (Number, %)				
No (0 g/day) Mild (0–20 g/day)	4568 (32.6) 180 (1.29)	302 (37.5) ^+^ 9 (1.12)	18,555 (76.4) 643 (2.65)	988 (84.7) ^+++^ 26 (2.23)
Moderate (≥20 g/day)	9253 (66.1)	494 (61.4)	5091 (21.0)	153 (13.1)
Coffee intake (Number %)				
Low (<3 g/day)	4660 (33.3)	308 (38.3) ^++^	10,883 (44.8)	645 (55.3) *^+++^
Medium (3–16 g/day)	9161 (65.4)	493 (61.2)	13,250 (54.6)	514 (44.0)
High (≥16 g/day)	180 (1.29)	4 (0.50)	156 (0.64)	8 (0.69)
Balanced diet pattern (Number, %)				
Low (<70th percentile)	9883 (70.6)	582 (72.3)	15,810 (65.1)	823 (70.5) ***
High (≥70th percentile)	4118 (29.4)	233 (27.7)	8479 (34.9)	344 (29.5)
Western-style diet pattern (Number, %)				
Low (<70th percentile)	10,012 (71.5)	623 (77.4) ***	19,869 (81.8)	989 (84.8) *
High (≥70th percentile)	3989 (28.5)	182 (22.6)	4420 (18.2)	178 (15.3)
Rice-based diet pattern (Number, %)				
Low (<70th percentile)	10,009 (71.5)	592 (73.5)	19,456 (80.1)	993 (85.1) ***
High (≥70th percentile)	3992 (28.5)	213 (26.5)	4833 (19.9)	174 (14.9)

Values represent adjusted means ± standard deviations after adjusting for covariates or the number of the subjects and percentage. Covariates used were age, gender, residence area, survey year, smoking, alcohol, education, job, income, energy, and physical activity. ^a,b,c,d^ Different letters indicate significant differences among the groups in the Tukey test at *p* < 0.05. * Significantly different for cataract incidence by two-way ANOVA in continuous variables at *p* < 0.05, ** at *p* < 0.01, and *** at *p* < 0.001. ^+^ Significantly different for gender by two-way ANOVA in continuous variables at *p* < 0.05, ^++^ *p* < 0.01, and ^+++^ at *p* < 0.001. HbA1c, blood hemoglobin A1c; LDL, low-density lipoprotein; HDL, high-density lipoprotein.

**Table 2:** The characteristics of the ten genetic variants of genes related to crystallin protein production, degradation, and aggregation in age-related cataract risk used for the generalized multifactor dimensionality reduction analysis.

Chr ^a^	SNP ^b^	Position	Mi ^c^	Ma ^d^	OR ^e^	*p*-Value Adjusted ^f^	MAF ^g^	*p*-Value for HWE ^h^	Gene	Functional Location
3	rs1417380362	41898108	C	T	0.770	1.13 × 10^−5^	0.1177	0.5644	*ULK4*	intron
5	rs117418426	150398496	G	A	1.648	5.74 × 10^−5^	0.01404	0.8807	*GPX3*	intron
7	rs200053781	8250586	T	G	0.859	5.55 × 10^−5^	0.3477	0.9565	*ICA1*	intron
7	rs147082589	97954290	C	T	1.684	1.26 × 10^−5^	0.01497	1.0	*BAIAP2L1*	intron
7	rs322348	136992106	C	A	0.700	4.77 × 10^−5^	0.05569	0.9063	*PTN*	intron
9	rs553983141	131368777	G	T	1.493	9.28 × 10^−5^	0.02212	0.6329	*SPTAN1*	intron
10	rs117583209	105320759	G	A	1.658	3.76 × 10^−5^	0.01418	0.3722	*NEURL1*	intron
11	rs2070894	111780837	G	A	0.837	8.61 × 10^−5^	0.2054	0.3761	*CRYAB*	intron
17	rs55785344	31914770	T	C	1.211	9.44 × 10^−5^	0.1311	0.9711	*ACCN1*	upstream transcript
17	rs879419608	71159820	C	T	0.804	5.78 × 10^−5^	0.1367	0.1619	*SSTR2*	upstream transcript

^a^ Chromosome; ^b^ Single nucleotide polymorphism; ^c^ Minor allele; ^d^ Major allele; ^e^ Odds ratio (OR) for age-related cataract; ^f^ *p*-value for OR after adjusting for age, gender, residence area, survey year, body mass index, daily energy intake, education, and income; ^g^ Minor allele frequency; ^h^ Hardy–Weinberg equilibrium.

**Table 3:** Generalized multifactor dimensionality reduction (GMDR) results of multi-locus interaction with genes related to age-related cataracts.

Genetic Model	Adjusted for Sex, Age				Adjusted for Sex, Age, Residence Area, BMI, Survey Year			
	TRBA	TEBA	*p*-Value	CVC	TRBA	TEBA	*p*-Value	CVC
*CRYAB*_rs2070894	0.5236	0.5142	8 (0.055)	7/10	0.5237	0.5143	8 (0.055)	7/10
*ULK4*_rs1417380362 *SSTR2*_rs879419608	0.5345	0.5206	9 (0.011)	7/10	0.5345	0.5206	9 (0.011)	7/10
*ULK4*_rs1417380362 *ACCN1*_rs55785344 *SSTR2*_rs879419608	0.5393	0.5175	8 (0.055)	3/10	0.5393	0.5175	8 (0.055)	3/10
*ULK4*_rs1417380362 *CRYAB*_rs2070894 *ACCN1*_rs55785344 *SSTR2*_rs879419608	0.5479	0.5228	8 (0.055)	6/10	0.5479	0.5228	8 (0.0547)	6/10
*PTN*_rs322348 plus model 4	0.5581	0.5247	10 (0.001)	10/10	0.5581	0.5248	10 (0.001)	10/10
*ICA1*_rs200053781 plus model 5	0.5673	0.5292	10 (0.001)	10/10	0.5673	0.5283	10 (0.001)	10/10
*BAIAP2L1*_rs147082 plus model 6	0.5743	0.5257	10 (0.001)	7/10	0.5743	0.5263	10 (0.001)	7/10
*SPTAN1*_rs553983141 plus model 7	0.5808	0.5215	9 (0.011)	5/10	0.5807	0.5243	9 (0.011)	5/10
*NEURL1*_rs11758320 plus model 8	0.5873	0.5274	10 (0.001)	10/10	0.5873	0.5272	10 (0.001)	10/10
*GPX3*_rs117418426 plus model 9	0.5922	0.5277	9 (0.011)	10/10	0.5922	0.5281	9 (0.011)	10/10

TRBA, trained balanced accuracy; TEBA, test balance accuracy; CVC, cross-validation consistency; *p*-value for the significance of the GMDR model by sign test adjusting for assigned covariates.

**Table 4:** Adjusted odds ratios for the age-related cataract risk by polygenetic risk scores of the best model (PRS) in the gene–metabolic syndrome after covariate adjustments.

Groups	Low-PRS (*n* = 2295)	Medium-PRS (*n* = 29,067)	High-PRS (*n* = 8949)	Genetic Variant–MetS Interaction *p*-Value
Middle-aged Elderly ^a^	1	2.07 (1.27–3.38) 1.43 (0.87–2.35)	2.92 (1.76–4.84) 2.03 (1.21–3.42)	< 0.0001
Men Women	1	1.98 (1.09–3.59) 1.63 (1.06–2.50)	2.48 (1.51–5.11) 2.35 (1.50–3.67)	0.813
Without MetS With MetS	1	1.86 (1.22–2.84) 1.47 (0.80–2.72)	2.81 (1.82–4.34) 1.74 (0.91–3.32)	0.715
Normal waist High waist ^b^	1	1.75 (1.23–2.47) 1.59 (1.07–2.38)	2.48 (1.73–3.55) 2.53 (1.67–3.83)	0.279
Normotension Hypertension ^c^	1	1.73 (1.10–2.71) 1.76 (1.03–3.04)	2.38 (1.49–3.80) 2.61 (1.03–3.04)	0.030
Low serum glucose High serum glucose ^d^	1	1.91 (1.28–2.87) 1.35 (0.70–2.58)	2.78 (1.83–4.21) 1.86 (0.92–3.79)	0.042

Values represent adjusted odds ratios and 95% confidence intervals. MetS, metabolic syndrome; waist, waist circumferences. PRS, including 6 SNPs selected from GMDR, was divided into three categories (0–6, 7–9, and ≥10) by tertiles as the low, medium, and high groups, respectively. The cutoff points of the parameters were as following: ≥65 years old ^a^, ≥90 cm for men and ≥85 cm for women ^b^, ≥130 mmHg systolic blood pressure (SBP) and ≥90 mmHg diastolic blood pressure (DBP) ^c^, and ≥126 mg/dL serum glucose concentrations plus hypoglycemic medicine ^d^. Multiple regression models included the corresponding main effects, interaction terms of gene and main effects, and potential confounders such as age, gender, residence area, survey year, smoking, alcohol, education, job, income, physical activity, hypertension, serum glucose concentrations, HbA1c contents, energy intake, percent intakes of fat, protein, and carbohydrate, and use of arthritis and dermatitis medications. Reference was the low-PRS.

**Table 5:** Adjusted odds ratios for the age-related cataract risk by polygenetic risk scores of the best model (PRS) in the gene–diet interactions after covariate adjustments.

Groups	Low-PRS (*n* = 2295)	Medium-PRS (*n* = 29,067)	High-PRS (*n* = 8949)	Genetic Variant–MetS Interaction *p*-Value
Low Na intake High Na intake ^a^	1	1.85 (1.54–3.70) 1.52 (0.83–5.01)	1.58 (1.14–3.50) 2.68 (1.43–5.01)	0.016
Low coffee intake High coffee intake ^b^	1	1.77 (1.05–3.00) 1.71 (1.08–2.72)	2.93 (1.71–5.04) 2.15 (1.33–3.45)	0.049
Low BD intake High BD intake ^c^	1	1.75 (1.24~2.48) 1.67 (1.13–2.47)	2.48 (1.73~3.55) 2.16 (1.44–3.24)	0.648
Low WD intake High WD intake ^d^	1	1.75 (1.23–2.47) 1.20(0.27–5.27)	2.27 (0.50–10.3) 2.48 (1.73–3.55)	0.049
Low RD intake High RD intake ^e^	1	1.75 (1.24–2.48) 2.04 (1.34–3.12)	2.48 (1.73–3.55) 2.94 (1.90–4.54)	0.146

Values represent adjusted odds ratios and 95% confidence intervals. BD, balanced diet; WD, Western-style diet consuming mainly noodles, bread, and red meat; RD, rice-based diet. PRS, including 6 SNPs selected from GMDR, was divided into three categories (0–6, 7–9, and ≥10) by tertiles as the low, medium, and high groups, respectively. The cutoff points of the parameters were as follows: ≥1600 mg/1000 kcal Na ^a^, ≥3 g/day coffee ^b^, a balanced diet pattern (>70th percentile) ^c^, Western-style diet including high intake of noodles, bread, and meat (>70th percentile) ^d^, and a rice-based diet pattern (>70th percentile) ^e^. Multiple regression models include the corresponding main effects, interaction terms of gene and main effects, and potential confounders such as age, gender, residence area, survey year, smoking, alcohol, education, job, income, physical activity, hypertension, serum glucose concentrations, HbA1c contents, energy intake, fat, protein, and carbohydrate percent intake, and arthritis and dermatitis medicine intake. Reference was the low-PRS.

## Supplementary Materials

The following are available online at https://www.mdpi.com/2072-6643/12/11/3534/s1, Table S1: Factor loadings of food groups in dietary patterns identified using principle component analysis, Table S2: Adjusted odds ratios for age-related cataract according to the polygenetic risk scores of the best model (PRS) for gene-gene interaction after covariate adjustments.
