# Supplementary material for: Polygenetic-Risk Scores Related to Crystallin Metabolism Are Associated with Age-Related Cataract Formation and Interact with Hyperglycemia, Hypertension, Western-Style Diet, and Na Intake
Source: Nutrients. 2020 Nov 17;12(11):3534. doi: 10.3390/nu12113534 (PMC7698476; doi:10.3390/nu12113534)
Supplement: Supplementary file 1 [file nutrients-12-03534-s001.pdf]

nutrients

Supplementary Material

Table S1. Factor loadings of food groups in dietary patterns identified using principle component analysis.

Food group	Balanced diet		Western-style diet		Rice-based diet	
Rice	-7		-5		90	*
Grain	6		6		-86	*
Noodles	-1		61	*	9	
Breads	10		57	*	-7	
Cakes	9		33		2	
Cookies	53	*	7		-4	
Beans	48	*	11		-9	
Potatoes	42	*	-1		5	
Kimchi	30		19		-2	
Eggs	1		77	*	1	
Fast foods	79	*	2		0	
Green vegetables	74	*	4		7	
Mushroom	61	*	-2		-4	
White vegetables	56	*	13		5	
Fatty fish	64	*	14		8	
White fish	42	*	19		11	
Crabs	23		8		-1	

Processed meats	32		38		21
Red meats	12		66	*	5
Soups	23		37		15
Chickens	59	*	2		-6
Seaweeds	36		7		-8
Milk	33		9		0
Beverages	-1		13		20
Coffee	5		21		20
Tea	42	*	0		-13
Fruit	41	*	4		10
Pickle	-1		11		16
Nuts	26		12		-15
Variance Explained by Each Factor	4.46		2.35		1.85

Printed values are multiplied by 100 and rounded to the nearest integer. *Values greater than 0.4.

Table S2. Adjusted odds ratios for age-related cataract according to the polygenetic risk scores of the best model (PRS) for gene-gene interaction after covariate adjustments.

	**Model 1**			**Model 2**	
	Low-PRS (*n* = 6,877)	Medium-PRS (*n* = 23,800)	High-PRS (*n* = 9,784)	Medium-PRS (*n* = 23,800)	High-PRS (*n* = 9,784)
BMI	1	0.95 (0.90-1.01)	0.97 (0.90-1.03)	0.93 (0.85-1.01)	0.97 (0.88-1.07)
Fasting serum glucose (>126 mg/dL)	1	1.05 (0.95-1.16)	1.11 (0.99-1.24)	1.07 (0.91-1.26)	1.13 (0.95-1.35)
HbA1c	1	1.18 (0.96-1.45)	1.17 (0.94-1.46)	1.14 (0.93-1.41)	1.12 (0.90-1.40)
Hypertension	1	1.01 (0.95-1.08)	1.01 (0.93-1.08)	1.04 (0.96-1.14)	1.04 (0.94-1.15)
Plasma LDL cholesterol	1	0.99 (0.92-1.07)	0.98 (0.91-1.07)	0.98 (0.89-1.08)	1.01 (0.90-1.13)
Plasma HDL cholesterol	1	1.03 (0.97-1.09)	1.03 (0.95-1.10)	1.05 (0.96-1.15)	1.08 (0.98-1.20)
Plasma TG	1	0.96 (0.91-1.02)	0.95 (0.89-1.02)	0.92 (0.85-1.01)	0.94 (0.85-1.03)

Values represent odd ratios and 95% confidence intervals. PRSBM including 5 SNPs selecting from GMDR was divided into 3 categories (0-5, 6-7, and ≥8) by tertiles as the low, medium and high groups, respectively. Low-PRSBM was the reference for both model 1 and model 2. *Significantly different from low PRSBM in logistic regression analysis at * $P<0.05$, ** $P<0.01$, *** $P<0.001$. Model 1: adjusted for age, gender, residence area, survey year, body mass index (BMI), education, job, and income. Model 2: adjusted for age, gender, residence area, survey year, smoking, alcohol, education, job, income, energy, activity, hypertension, milk, fat percent intake, carbohydrate percent intake, and arthritis and dermatitis medicine intake.

© 2020 by the authors. Submitted for possible open access publication under the terms and conditions of the Creative Commons Attribution (CC BY) license (http://creativecommons.org/licenses/by/4.0/).
